# Quantum Chemical Computation of Omicron Mutations Near Cleavage Sites of the Spike Protein

**DOI:** 10.3390/microorganisms10101999

**Published:** 2022-10-10

**Authors:** Puja Adhikari, Bahaa Jawad, Rudolf Podgornik, Wai-Yim Ching

**Affiliations:** 1 Department of Physics and Astronomy, University of Missouri-Kansas City, Kansas City, MO 64110, USA; 2 Department of Applied Sciences, University of Technology, Baghdad 10066, Iraq; 3 School of Physical Sciences and Kavli Institute of Theoretical Science, University of Chinese Academy of Sciences, Beijing 100049, China; 4 CAS Key Laboratory of Soft Matter Physics, Institute of Physics, Chinese Academy of Sciences, Beijing 100090, China; 5 Wenzhou Institute of the University of Chinese Academy of Sciences, Wenzhou 325000, China

**Keywords:** SARS-CoV-2, Omicron variant, spike protein, cleavage sites

## Abstract

The attachment of the spike protein in SARS-CoV-2 to host cells and the initiation of viral invasion are two critical processes in the viral infection and transmission in which the presence of unique furin (S1/S2) and TMPRSS2 (S2′) cleavage sites play a pivotal role. We provide a detailed analysis of the impact of the BA.1 Omicron mutations vicinal to these cleavage sites using a novel computational method based on the amino acid–amino acid bond pair unit (AABPU), a specific protein structural unit as a proxy for quantifying the atomic interaction. Our study is focused mainly on the spike region between subdomain 2 (SD2) and the central helix (CH), which contains both S1/S2 and S2’ cleavage sites. Based on ab initio quantum calculations, we have identified several key features related to the electronic structure and bonding of the Omicron mutations that significantly increase the size of the relevant AABPUs and the positive charge. These findings enable us to conjecture on the biological role of Omicron mutations and their specific effects on cleavage sites and identify the principles that can be of some value in analyzing new variants.

## 1. Introduction

The SARS-CoV-2 virus continues to mutate and evolve, leading to major variants of concern (VOC) that can significantly change the virus characteristics [1]. These VOC result in increased infectivity, transmissibility, and severity as well as reduced efficacy of available therapies [1,2,3]. The Omicron variant (OV), the most recently identified VOC, rapidly became the dominant strain globally due to an unprecedented number of mutations in the spike (S) protein, making it highly contagious and/or vaccine-resistant [4,5,6]. During the early stages of virus infection, the S-protein mediates both the receptor binding via its S1 subunit as well as the membrane fusion via its S2 subunit. Upon binding of the S-protein to the human angiotensin-converting enzyme 2 (ACE2) in the host cell, the S-protein is proteolytically cleaved at two protease recognition sites: the S1/S2, or furin cleavage site, and the TMPRSS2 S’, or *transmembrane serine protease 2 cleavage site*, in order to activate the fusion machinery [7]. The furin S1/S2 site is located at the boundary between the S1 and S2 subunits, exhibiting a unique polybasic insertion furin recognition site _681_PRRAR|S_686_ (“|” denotes proteolytic cleavage site) [8], while the TMPRSS2 S’ site is located just upstream of the fusion peptide (FP) domain of the S2 subunit. These cleavage sites play an essential role in viral infectivity, transmissibility, fusogenicity, and pathogenicity [8,9]. Mutations at the cleavage sites have been shown to promote more efficient cell–cell fusion in both Alpha and Delta VOCs and can facilitate and enhance cell entry, thus increasing transmissibility [10,11]. Near the S1/S2 cleavage site, OV has the same mutation of P681H as the Alpha variant, connected to the increased infectivity, in addition to two other mutations of H655Y and N679K [12]. Moreover, OV BA.1 has six unique mutations at the S2 subunit (N764K, D796Y, N856K, Q954H, N969K, L981F) that have not been previously detected and whose biological functions are unknown [12]. Therefore, it is urgent to identify the role of these novel OV mutations and investigate their impact on the S-protein, particularly around the proximal region of the cleavage sites. This will provide information on the change in interatomic interaction and guide effective therapeutic strategies to overcome current and future cross-strain SARS-CoV-2 infections.

**Figure 1:**
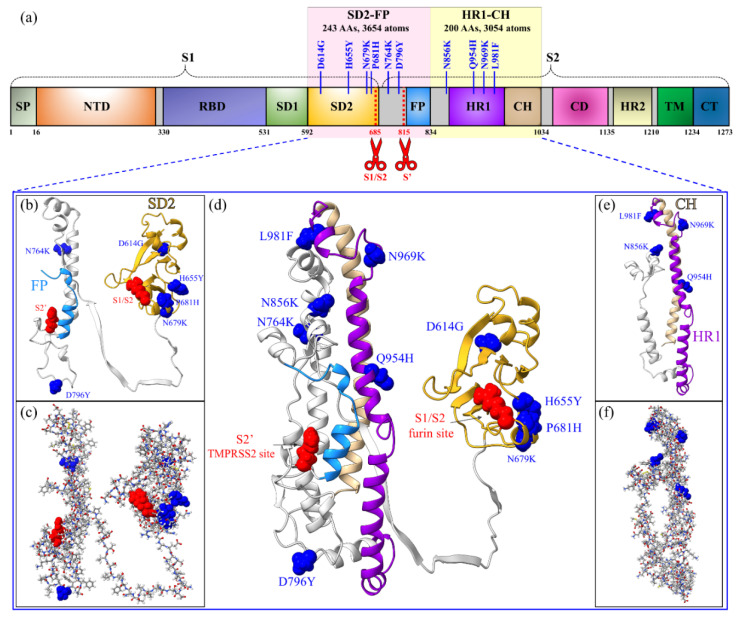
Graphic illustration of SD2-FP and HR1-CH models. (**a**) Schematic of S-protein primary structure divided into domains with cleavage sites S1/S2 and S2’ denoted by scissors and highlighting the SD2-FP and HR1-CH regions with their 10 Omicron mutations. (**b**,**c**) SD2-FP models in ribbon and ball and stick representations, respectively. Their six Omicron mutations are marked and shown by a blue sphere, while the S1/S2 and S2′ cleavage sites are represented by a red sphere. (**d**) Ribbon figure of SD2-FP and HR1-CH showing the two cleavage sites (S1/S2 and S2′) and marked the 10 Omicron mutations in these regions. (**e**,**f**) HR1-CH model with their four Omicron mutations in different representations. The red, blue, grey, yellow, and white in ball and stick figures are for O, N, C, S, and H atoms, respectively.

The S-protein of SARS-CoV-2 is the main target of most therapies [13,14,15,16]. It appears in the trimeric form, with each protomer comprising two functional subunits, S1 and S2, that are demarcated by a furin cleavage site (S1/S2), as shown in Figure 1. S1 contains the signal peptide (SP), N-terminal domain (NTD), receptor-binding domain (RBD), subdomain 1 (SD1), and subdomain 2 (SD2), while S2 consists of fusion peptide (FP), heptad repeat 1 (HR1), central helix (CH), connector domain (CD), heptad repeat 2 (HR2), transmembrane domain (TM), and cytoplasmic tail (CT) [17]. Viral entry into the host cell depends on these two subunits. The cleavage sites play a role in facilitating SARS-CoV-2 entry. The cleavage activation mechanism occurs at S1/S2 and S2ʹ, which is a very complex process [18]. Due to the complexity of the role played by the cleavage sites in virus transmission and the lack of information on this process of the Omicron variant, an accurate state-of-the-art ab initio computational study could reveal some intricate details related to the mutations close to these cleavage sites. 

In this work, we focus on the impact of the Omicron mutations close to the cleavage sites S1/S2 and S2′ between SD2 to FP (SD2-FP) and HR1 to CH (HR1-CH), which contains 10 mutations (D614G, H655Y, N679K, P681H, N764K, D796Y, N856K, Q954H, N969K, L981F) (see Figure 1). Most importantly, the last six mutations (N764K to L981F) in S2 are the unique mutations in OV. We constructed two models for SD2-FP and HR1-CH, one for the wild-type (WT) or unmutated case and the other one for the mutated OV BA.1, as shown in Figure 1d. Interestingly, eight of the 10 BA.1 OV mutations in these two models are shared by BA.2, BA.2.75, BA.3, BA.4, and BA.5, while the remaining two mutations, N856K and L981F, are unique to BA.1 OV. In other words, these two models could forecast the consequences of the eight common mutations found in other Omicron subvariants at the spike protein region between SD2 and CH. The two models must be calculated separately within the ultra-large first principles ab initio approach for biomolecules, based on the *divide and conquer strategy* [19,20]. The SD2-FP model has 3654 atoms (WT) and 3681 atoms (OV), and HR1-CH has 3054 (WT) and 3071 atoms (OV), including H atoms. Figure 1d illustrates the two parts together and will be elaborated in Section 2.1. We employ the novel concept of amino acid–amino acid bond pair (AABP) as specific protein structural unit [21] to quantify and characterize the details of the impact of OV mutations, which could help to determine their role in high infectivity.

## 2. Methods

### 2.1. Model Construction

For large-scale ab initio quantum chemical calculations of a complex biomolecular system, the first step is to design the starting atomistic model. Based on the *divide and conquer strategy* [19,20,21], SD2-FP and HR1-CH are constructed as separate models in wild-type (WT) and in mutated forms (OV), as depicted in Figure 1. SD2-FP and HR1-CH contain 243 and 200 amino acids, respectively. We followed the same approach, previously developed in the Delta variant simulation, to build these systems [20]. The initial structure for the SARS-CoV-2 S-protein shown in Figure 1 was obtained from Woo et al., 6VSB 1_2_1, from [PDB ID 6VSB] [22], which originated from the Wrapp et al. study [17]. We chose Chain A in its up conformation and used it to create the wild-type (WT) and mutated Omicron variant (OV) models. Sequence numbers for the SD2-FP model and HR1-CH model are 592–834 and 835–1034 in 6VSB 1_2_1 [23], respectively. This procedure is summarized as follows. First, we selected residues from 592 to 834 of the SD2 and FP regions to create the SD2-FP model. Next, we removed the glycans and the associated hydrogen (H) atoms from the SD2-FP model and later added the H atoms using the Leap module in the AMBER package [24,25], which then yields the unmutated or wild type (WT) model. This WT model is used as a template to generate the mutated OV model of SD2-FP with six substitutions: D614G, H655Y, N679K, P681H, N764K, and D796Y using the Dunbrack backbone-dependent rotamer library [26] implemented by the UCSF Chimera package [27]. Similar steps were followed to prepare the WT and OV models for HR1-CH. The HR1-CH OV model contains four mutations: N856K, Q954H, N969K, and L981F. The SD2-FP model has 3654 atoms (WT) and 3681 atoms (OV), and HR1-CH has 3054 (WT) and 3071 atoms (OV), including H atoms. All four models—one WT of each of SD2-FP and HR1-CH, and two relevant OV models—were first minimized with 500 steepest descent steps and 10 conjugate gradient steps using UCSF Chimera to avoid bad clashes of unrealistic close atomic pairs. These four initial atomistic models are further optimized to sufficient accuracy using VASP (see the next subsection). Figure S1 shows the optimized atomic-scale structure in the models, like the ribbon structure depicted in Figure 1d.

We would like to point out two limitations or challenges that could not be avoided during our research. The first is that extremely large-scale ab initio all-atom calculations of the entire S-protein are clearly impossible currently. Therefore, our study is limited to small, manageable size models, such as SD2-FP and HR1-CH, which include Omicron mutations at the S1/S2 and S2′ cleavage sites. Second, any significant conformational changes that could be associated with these ten OV mutations are difficult to assess using these small models and our ab initio approach. It is necessary to include all atoms of the S protein trimer, which is currently impossible to perform in a single QM calculation. Other approaches, such as molecular dynamics (MD) simulation, will be useful to investigate any conformational changes for the entire S-protein, which are beyond the scope of our current study.

### 2.2. Vienna Ab Initio Simulation Package (VASP)

In this study, we used two well-established density functional theory (DFT)-based packages: the Vienna ab initio Simulations package (VASP) [28] and the orthogonalized linear combination of atomic orbital (OLCAO) method [29], which we will describe in the next subsection. The combination of these two different DFT codes, VASP and OLCAO, has been successfully used in a variety of areas, including large biomolecules, organic or inorganic disordered systems, etc. [19,20,21,30,31,32,33,34,35,36].

The initial structures derived from the protein data bank (PDB) are further modelled as discussed in Section 2.1 and are fully relaxed by using VASP [28], which is known for its efficiency in structural optimization. A large cell is used so that the protein is around 15 Å apart to avoid periodic boundary conditions. The projector augmented wave (PAW) method with Perdew–Burke–Ernzerhof (PBE) exchange correlation functional [37] within the generalized gradient approximation (GGA) is one of the options we chose that balances the accuracy needed and the computational resources available. Detailed tests in the past suggest that the use of the following input parameters for biomolecular systems: (1) energy cut-off at 500 eV; (2) electronic convergence of 10^−4^ eV; (3) force convergence for ionic steps at −10^−2^ eV/Å; (4) single k-point sampling at the center of the supercell. The final relaxed structure of the models has been achieved with a total energy difference of less than −0.330 eV or −0.000108 eV per atom and will be used as the input for OLCAO calculations. 

### 2.3. Orthogonalized Linear Combination of Atomic Orbitals (OLCAO) Method

A different DFT method, the OLCAO method, developed in-house [29], is used to calculate the electronic structure and interatomic interactions of biomolecular systems. The OLCAO method uses minimal atomic basis expansion and orthogonalization to the core orbitals technique in order to deal with the huge matrix with a single diagonalization to obtain all the energy states and wave functions of the Kohn–Sham equation [29]. The key feature of the OLCAO method is the provision of two fundamental structural parameters: the effective charge ($Q^{*}$) on each atom and the bond order (BO) values *ρ_αβ_* between any pair atoms α and β in the large supercell of the biomolecule. They are obtained from the ab initio wave functions with atomic basis expansion, calculated quantum mechanically for the large biomolecule. The two OLCAO structural parameters are defined as:

(1) $$Q_{\alpha }^{*}=\underset{i}{\sum }\underset{m,occ}{\sum }\underset{j,\beta }{\sum }C_{i\alpha }^{*m}C_{j\beta }^{m}S_{i\alpha ,j\beta }\;$$

(2) $$\;\;\rho_{\alpha \beta }=\underset{m,occ}{\sum }\underset{i,j}{\sum }C_{i\alpha }^{*m}C_{j\beta }^{m}S_{i\alpha ,j\beta }\;$$

In the above equations, $S_{i\alpha ,j\beta }\;$ are the overlap integrals between the $i^{\mathrm{th}}$ orbital in the $\alpha^{th}\;$ atom and the $j^{\mathrm{th}}$ orbital in the $\beta^{\mathrm{th}}\;$ atom. $C_{j\beta }^{m}$ are the eigenvector coefficients of the $m^{th}$ occupied molecular orbital. The deviation of $Q_{\alpha }^{*}\;$ from the neutral atomic charge $Q_{\alpha }^{0}$ on the same atom  α is usually referred to as the partial charge (PC).

(3) $$\Delta Q_{\alpha }=Q_{\alpha }^{0}\;-Q_{\alpha }^{*}\;\;$$

From Equation (3), the PC value for every atom in the model can be calculated. This PC can be further unified for every AA (PC^AA^) as well as any desired unit. In this study, we have also analyzed partial charge for *amino acid amino–acid bond pair unit* (AABPU) denoted by PC*. PC* is discussed below in Section 3.1 and Section 3.3.

The BO quantifies the strength of the bond between two atoms in the unit of electrons (e^−^) and usually scales with the bond length (BL) or the distance of separation between atoms α and β, also depending on the configuration of the vicinal atoms. It should be pointed out that the calculated PC and BO in Equations (1) and (2) are basis-dependent since they are based on the Mulliken scheme [38,39] using localized atomic orbitals. We use the minimal basis in all our calculations for biomolecular systems. Another very important point is that we use a one-point calculation to obtain all BO values for all atomic pairs to characterize the internal cohesion of the system under study, rather than the traditional total energy or enthalpy calculation (two-point or even many-point calculation), which is used to describe the strength of binding between biomolecular systems. The sum of all BO values within a structural component—such as a protein—gives the total bond order (TBO), which accurately describes the interatomic interactions that are critical to analyzing the AA–AA network for large complex biomolecules. 

In complex biomolecules, one can extend the concept of the bond order (BO), defined for a pair of atoms, and apply it to the interaction between a pair of amino acids (AAs). We refer to this generalized quantifier of molecular interactions as the *amino acid–amino acid bond pair* (AABP) [32], defined as:

(4) $$AABP\left(u,v\right)=\underset{\alpha \epsilon u}{\sum }\underset{\beta \epsilon v}{\sum }\rho_{\alpha i,\beta j\;}\;$$

In Equation (4), the summations are over all atoms α in AA u and all atoms β in AA v. AABP considers all possible bonds between two AAs, including both covalent and hydrogen bonding (HB). AABP value is a single parameter proxy that quantifies the interaction between two AAs. The stronger the interaction, the higher the AABP will be and vice versa. AABP can be further resolved in nearest neighbor (NN-AABP) and non-local (NL-AABP) parts, which are discussed in detail in Section 3.1. It should be emphasized that the AABP does not involve the “BL” used for the description of interacting atoms since the distance of separation between two AAs is difficult to quantify precisely. AABP values are calculated from quantum mechanical wave functions of the entire biomolecular system and thus represent a collective structural parameter, including the effects of all atomic pairs involved. AABP is a new fundamental measure of molecular interactions in biomolecules that contains the nearest-neighbor or local interactions of AAs that are vicinal along the sequence and in the 3D folding space, as well as the off-diagonal or non-local interactions between AAs that are not vicinal in the sequence space but are interacting in 3D folding space.

## 3. Results 

### 3.1. Amino Acid—Amino Acid Bond Pair (AABP) Unit

In the Methods section, we have described the previously introduced AABP [32] as an extension of the AA–AA interaction description that also includes the contribution from non-local AAs that are not vicinal along the primary AA sequence. The AABP considers all possible bonds between two AAs, including both covalent and hydrogen bonding (HB). This single quantifier, derived directly from ab initio quantum chemical calculations for large supercells of several thousands of atoms, reflects the internal bonding strength between all relevant amino acids. Such information is essential for analyzing the effects of mutations on the properties of a protein as well as the whole virus. AABP can be further resolved into nearest neighbor (NN) bonding and non-local (NL) bonding from non-NN pairs, including HBs along the 3D protein structure. In this sense, AABP is an ideal parameter to characterize interactions between different AAs or groups of interacting AAs in biomolecules, i.e., we can consider AABP to characterize a specific structural unit in protein: the AABP unit (AABPU) that has been thoroughly described in reference [21]. Hence, the AABPU is capable of providing full physical insight into properties such as interatomic interactions, partial charge distributions, size, shape, NL–AA interactions, etc.

The results for the structure and some pertinent properties of the 10 mutations in the two models containing the cleavage sites are summarized in Table 1. The salient features of the results in Table 1 are summarized as follows:

(1) The largest total AABP is from site 796, close to cleavage site S2′, with only two NL–AAs. D796Y mutation reduces AABP slightly, but it increases size, surface exposure, and positive charge. The largest NN–AABP is from the same site 796 with only two NL–AAs. This confirms that *NN–AA is the dominant interaction,* as evidenced by the primary sequence of the S-protein.
(2) The smallest total AABP comes from site 981, which is relatively far from the S′ cleavage site but near the prefusion-stabilizing two-proline (2P) mutations (K986P and V987P) utilized in Pfizer-BioNTech and Moderna vaccines [13,14]. Site 981 also has the smallest NN–AABP in WT but with NL–AAs of 5.
(3) The 954 site has the largest NL–AABP contribution among all 10 mutations. It is part of the HR1 domain, as are the other two sites, 969 and 981, which have low NL–AABPs. These three Omicron mutations have slightly different interatomic interactions than their WT counterparts, while their size, shape, and PC*s are all changed. We speculate that these mutations may play a role in the binding of HR1 and HR2 domains to enhance the formation of six-helix-bundle (6-HB), which brings the viral lipid and the host lipid membranes close together, resulting in membrane fusion and the initiation of infection [40]. Here, it should be mentioned that the HR1-CH model alone is insufficient to assess this binding process. All atoms of the post-fusion S-protein must be included, which is currently impossible to do in a single ab initio calculation.
(4) The smallest NL–AABP is from site 614 in group A ahead of the S1/S2 cleavage site. D614G mutation reduces the number of NL–AAs from 5 to 3 but results in a shift in charge distribution toward a more positively charged state, which could enhance the susceptibility of protease cleavage at the S1/S2 junction and/or promote the up conformation of the S-protein, as previously reported [41,42,43].
(5) The largest contribution from HB to total AABP is from site 954, while the smallest HB contribution is from sites 614 and 655. This attests to the importance of the contribution of HB to the overall bonding network.
(6) The role of the PC* distributions is obvious from five mutations, D614G, N679K, P681H, D796Y, and N969K. They exhibit a significantly changed, more positive, PC*. Importantly, the mutation at the 681 site, which is adjacent to the furin cleavage site, has been reported to play a significant function in the cleavage process [10,11,20,44]. Increasing the positive charge of P681H is necessary for the host furin-like proteases to cleave the S-protein [11]. Additionally, the N679K mutation is also located in the furin cleavage region and has been reported to increase the furin-mediated cleavage of Omicron [45]. However, some investigations have indicated that these N679K and P681H mutations do not enhance the S-protein cleavage processing and may even be less efficient [12,46,47,48]. This suggests that additional mutations near the furin cleavage site may interfere with its cleavage.

These observations clearly indicate that the changes in the total AABP values and their non-local components depend on the nature of the substitution, interatomic interactions, location with respect to the cleavage sites, and the HB contribution. 

**Figure 2:**
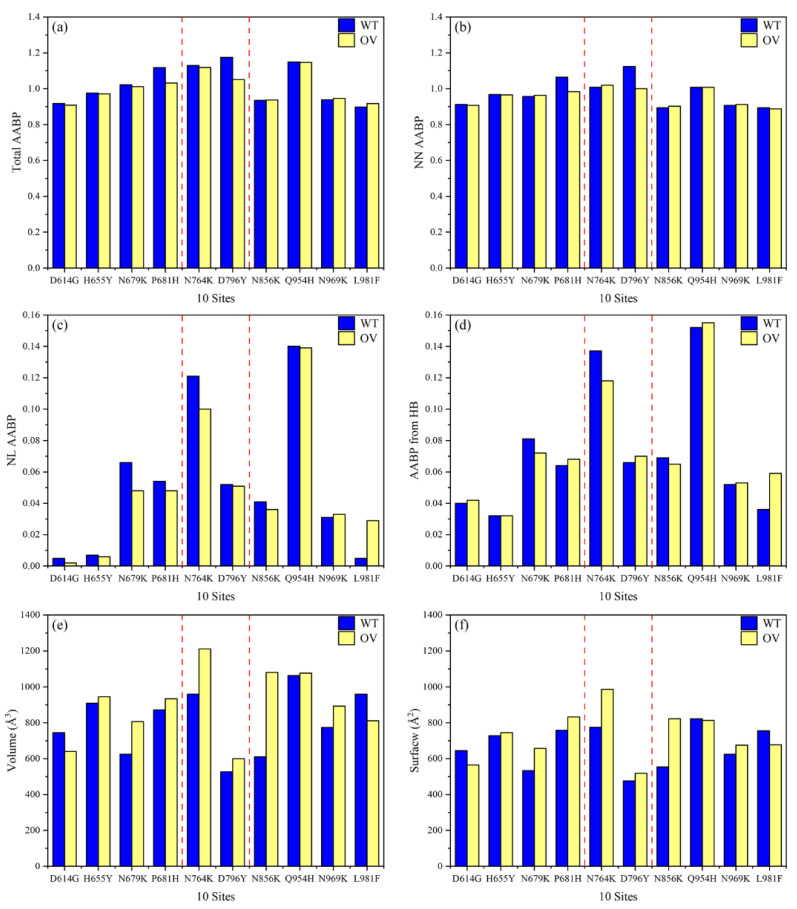
Comparison of interatomic interactions and geometry features of 10 OV mutations with their corresponding WT. (**a**) Total AABP, (**b**) NN AABP, (**c**) NL AABP, and (**d**) AABP from HB, for 10 unmutated (WT) and mutated (OV) AAs. (**e**) Volume, and (**f**) Surface. The two dashed red lines show the two cleavage sites.

In Figure 2, we plot the main results of Table 1 with the histogram bars, showing the changes due to mutations side by side. 

**Figure 3:**
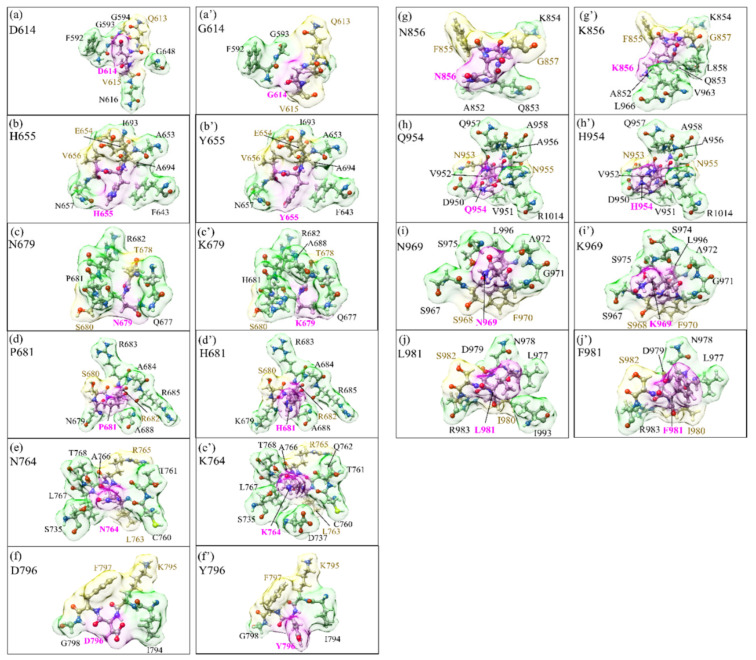
Distribution of the six mutation sites in SD2-FP (**left** panel) and four mutation sites in HR1-CH (**right** panel). Within each panel, (**a**–**j**) on the left column is for the WT, and (**a′**–**j′**) on the right column is for the OV. The surface of mutated sites is shown in magenta, surface of NN and NL are shown in yellow and green, respectively. All NN and NL AAs are marked near to their surface in brown and black, respectively. The geometrical scale is kept the same in all figures.

Figure 2 essentially reflects the observations listed above. The distribution of the total AABP values in (a) is closely mimicked by the NN–AABP values in (b), reflecting the fact that the AA sequence is always the most relevant ordering in proteins, but the addition of NL–AABP is not negligible, as shown in (c), and accounts for the majority of HB contributions to the total AABP, as illustrated in (d). An important accomplishment is that we can calculate the volume and surface areas of each AABPU as a specific structural unit. Another important observation from Figure 2 is that mutations slightly decrease the AABP values in most cases, but they tend to increase the volume of AABPU, some of them rather substantially (N764K and N856K). The plots for the AABPU volume and surface area in (e) and (f) mimic each other as expected but do not coincide, since the complexity of the shapes of the AABPU plays a role that defies simple quantification. This will be illustrated in Figure 3 and Figure S1 in the Supplementary Materials (SM) to follow. Out of 10 mutations, only two have decrease in volume, D614G at far left and L981F at far right. 

Figure 3 displays the mutation effect in 2D plots for the 3D structures with the same length scale. The volume and surface data in Table 1 provide the geometry of the Omicron pictorially by connecting the mutation effect with the proximity to the cleavage locations. With the exception of D16G and L981F, OV mutations tend to increase their volume. N856K, which is located between the FP and HR1 domains, has the greatest change in volume. While the smallest change in volume comes from Q956H. 

To better illustrate the size and shape due to mutation, we replot the cases of the N856K and Q954H mutations in more detail in Figure S2a,b, respectively, with corrected length and scale and in three different orientations. As shown in Table 1, N856K has the largest increase in volume of 77.2% and the shape of the AABPU is drastically different between WT and OV. On the other hand, Q954H has the largest volume both before and after mutation, so the difference in the increased volume is small, with only 1.3%. We also replotted similar figures for N764K and D796Y mutations in Figure S2c,d, respectively. Both N764K and D796Y are between the two cleavage sites S1/S2 and S2′ at the N-terminal of the S2 subunit and both increased their volume by 26.2% and 13.7%, respectively. The biological function of the D796Y mutation is still unclear. Previously, it has been revealed that the mutation at this site impairs the neutralization susceptibility of antibodies and reduces SARS-CoV-2 infectivity [49]. Based on detailed ab initio calculations, we expect that shifting the partial charge of the N764K and D796Y toward more positive values as their sizes increase could impact the cleavage site S′, the interactions between S1 and S2 subunits, or the susceptibility to neutralizing antibodies. Further investigations are necessary here. 

### 3.2. Interatomic Bonding

The total and partial density of states (TDOS and PDOS) for SD2-FP and HR1-CH in both cases have been shown in Figures S3 and S4 with many interesting observations that are fully described in SM. 

**Figure 4:**
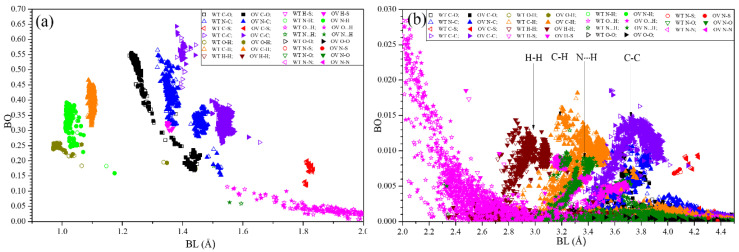
Overlap distributions of BO vs. BL for the WT and OV SD2-FP and HR1-CH models with (**a**) 0.9 Å to 2.0 Å BL and (**b**) 2.0 Å to 4.5 Å. WT and OV are shown in open and closed symbols, respectively.

Figure 4 shows the distribution of bond order (BO) vs. bond length (BL) for all atomic pairs in the models SD2-FP and HR1-CH with BL ranging from (a) 0.5 Å to 2.0 Å and (b) 2.0 Å to 4.5 Å. Such large atomic-scale calculation is truly unprecedented and can reveal many of the intricate details in bonding close to the location of the cleavage sites S1/S2 and S2′. As can be seen, mutations do slightly shift the positions of the data points. However, obtaining details of specific bonding pairs close to the cleavage site is a daunting task. Most of the data points in Figure 4a with large BO values are from stronger covalent bonds. Those unaffected by mutations are strictly the internal covalent bonds within the AAs. 

We would like to focus on the HBs with BL above 1.56 Å and the data are shown as star symbols. The strongest HB has a BO of 0.12 e- at 1.56 Å. Mutation can either enhance or weaken the HB strengths. Even though HBs are relatively weak, they are ubiquitous and constitute a significant component of interatomic bonding in biomolecules (see Figure 4b). Figure 4b also shows data points of bonds at separations above 2.0 Å. There are four main groups of bonds: H-H, C-H, N∙∙∙H and C-C, indicated in the middle part of Figure 4b. They all indicate that for an accurate description of interatomic bonding and the effect of mutation, interactions for atomic pairs up to at least 4.5 Å are necessary. 

In Figure 4a, near 1.16 Å the covalent N-H bond has BO lowered by mutation. At 1.39 Å, the covalent O-H bond, mutation makes a negligible difference. At 1.52 Å, for the covalent N-C bond, mutation slightly lowered the BO. Near 1.63 Å, in the covalent C-C bond, mutation increased the BO. In Figure 4b at 2.49 Å, the covalent H-S bond increased BO due to mutation. For HBs in both Figure 4a,b, mutation can both increase and/or decrease the BO by a small amount. All this indicates that at the atomistic level, mutations can increase or decrease the bond strength, a situation correctively reflected in the column AABP (HB) of Table 1. Quantitatively, the total number of N∙∙∙H and O∙∙∙H HBs in the SD2-FP model for WT (OV) is 1285 (1285) and 2080 (2091), respectively, indicating an increase in O∙∙∙H but no change in N∙∙∙H. On the other hand, the number of HBs in the HR1-CH model for WT (OV) is 1039 (1063) for N∙∙∙H and 1721 (1720) for O∙∙∙H, showing a change in N∙∙∙H but one less HB in O∙∙∙H of OV. This analysis reveals that the intramolecular HBs alter slightly due to OV mutations.

### 3.3. Partial Charge

**Figure 5:**
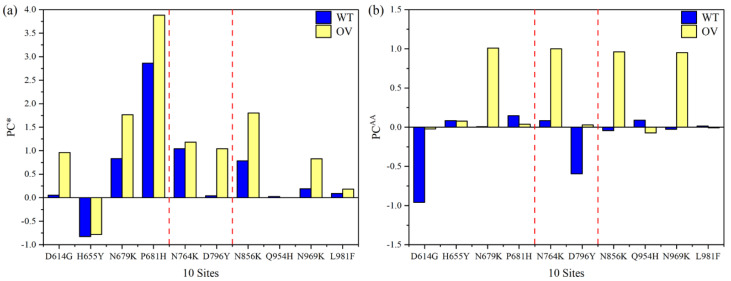
Partial charge distribution. (**a**) Partial charge per AABPU for 10 mutations, WT (blue) and OV (yellow). The two dashed red lines show the two cleavage sites. (**b**) PC^AA^ is the partial charge distribution of the AA at each unique site.

Figure 5a shows the PC* distribution of the AABPU listed in Table 1 for the 10 mutations. It is really striking to see that nearly all the PC* on Omicron are positive. The only mutation with a negative PC* is H655Y. The other nine mutations, or 90%, have positive PC* and all exhibit a significant increase in PC* after mutation. This is a very important result obtained from our ab initio DFT calculation and has profound implications since the cell membranes in the human body are mostly negatively charged [50]. Therefore, these mutated AAs will have enhanced non-specific electrostatic interactions with the cell membrane units. The shift toward positive PC* of OV mutations has a direct impact on the cleavage sites (indicated by vertical red dotted lines), especially the large increases in N679K and P681H, which are very close to the furin S1/S2 site, and notable increases between D796Y and N856K that are surrounding the S′ site. This increase in the number of positive charges is crucial for the host protease to cleave the S-protein. However, several experimental studies have found that the Omicron S-protein cleaves less efficiently than other VOCs [12,30,31,32], while another study has indicated the opposite [45]. This contradiction indicates that increased PC in Omicron may have other consequences not yet elucidated. For example, N764K, N856K, and N969K mutations have been observed to form interprotomer electrostatic contacts with the neighboring protomers, enhancing S-protein stability [51]. 

The trend toward positive charges of OV mutations is emphasized further in Figure 5b, detailing the PC^AA^ of the key AA at each site. To easily distinguish them, we used two PC symbols: PC* for the whole unit of AABPU and PC^AA^ for only one unique AA site. Interestingly, the D614G, N679K, N764K, D796Y, N856K, and N969K OV mutations significantly shift their PC^AA^s to positive, whereas H655Y, Q954H, and L981F have minor or negligible changes. P681H exhibits a distinct behavior when comparing Figure 5a,b, since the substitution of P to H changes the PC* of AABPU rather than the key AA itself. The reason could be connected to the fact that we performed our simulation at neutral pH, while the pH impact could play a significant role in the cleavage process, particularly regarding the charge of 681 site.

## 4. Discussion

Protein–protein interactions (PPIs) are essential for many biological processes, so they have to be accurately identified not only at the molecular level but also at the AA and atomic levels. In fact, the AA–AA pairing governs these PPIs as well as protein folding and assembly. In this paper, we strongly advocate the concept of AABPU, based on the AABP interaction proxy, as a special structural unit to describe and quantify atomistic level resolution of biomolecular structures and interactions. AABPU includes many key parameters, such as interatomic interaction, PC, size, and shape. AABPU may be useful for studying biological networks ranging from AA–AA pairing to 3D protein structure and protein–protein networks. It provides new insights into the geometry alterations and interatomic interactions of different S-protein domains. This concept could also be extended to investigate the mutational effects on other S-protein domains and S-protein protomers not covered in this study. 

The cleavage efficiency of S-protein at the furin-cleavage S1/S2 site increases the chances that the newly generated virus will be able to fuse rapidly with the host membrane. In this regard, mutations in the Alpha and Delta variants′ S1/S2 junction region have been shown to improve the cleavage efficiency at this site [10,11]. Specifically, the Delta variant had a higher transmission rate than Alpha, owing to its P681R mutation vs. P681H mutation of Alpha [44]. Omicron, like Alpha, has a P681H mutation, so its cleavage efficiency may be less than that of the Delta variant, as recently reported [12]. Additionally, Omicron bears two more mutations close to this site, H655Y and N679K, which are likely to impact its S-protein cleavability by adding more positive charges to this site, but there are many debates about this prediction. Some studies have found that Omicron S-protein cleaves less effectively than other variants [12,46,47,48], while others have revealed the opposite view [45].

The cell–cell fusion also requires the S′ site. Protease activity can occur either by TMPRSS2 at the plasma membrane or by cathepsins in the endosomes [7]. TMPRSS2 activity has been established to be critical for viral spread and pathogenesis in the infected host [7]. Indeed, it has been claimed that TMPRSS2 is necessary for optimal virus entry in both the WT and Delta variants, but not in Omicron [12]. This suggests that Omicron prefers the endocytic cell entry route instead of the plasma membrane entry route [12,47,48]. This is consistent with the idea that reduced cleavage efficiency of Omicron S-protein at the S1/S2 site impairs TMPRSS2-mediated entry [12].

Together, the roles of the cleavage sites of Omicron are still unclear and requires further investigation. The main goal of this work is to speculate on the connection between Omicron mutations at proximal cleavage sites and the cleavability and fusogenicity based on AABPU. It is clear from our study that the Omicron mutations induce more positive partial charges in the charge distribution of S-protein. This change may play a function in controlling proteolytic cleavage, render S-protein more sensitive to low-pH induced conformational changes, promote an adaptation that utilizes the low-pH endosomal entry pathway, and/or boost virus entrance in the lower pH environment of the upper airway [12]. 

Unlike the Delta variant, the high transmissibility of OV may not be due to its efficient cleavability, but rather to the immune evasion and the strong binding affinity between RBD and ACE2 [6,12,46,47,48]. Therefore, more studies are needed to explore the impact of Omicron mutations on neutralizing antibodies and ACE2 binding, and how they are responding to other Omicron subvariants, such as BA2 or BA3, for which we believe that the AABPU-based approach we developed can provide this valuable information.

Another future direction that has not been addressed above is how to use the calculated data on AABPU in Machine Learning (ML) applications, which is described in SM with Tables S1 and S2.

Finally, we would like to point out that *ab-initio* can complement molecular dynamics (MD). Even though there is a size limitation in *ab-initio* calculation, its pseudopotential can provide a valuable alternative to analyze complicated biological systems in comparison to a priori force fields of MD simulation. In our past calculation, PC obtained from *ab-initio* calculation for the RBD–ACE2 interface complex were fed into the MD force field for prediction of electrostatic interaction [30].

## 5. Conclusions

In summary, we analyzed both WT and OV models for SD2-FP and HR1-CH regions, including the cleavage sites S1/S2 and S2′ of S-protein. We developed a quantitative scheme and methods for rapid assessment of the effect of 10 mutations in OV using the novel concept of AABPU. Based on AABPU, we have further analyzed its interatomic bonding, volume, surface area, and PC*. We have conjectured a possible reason for the fast infection rate in the Omicron variant based on this detailed analysis. This ab initio study provides a road map for computational research on new variants of concern in the SARS-CoV-2 virus.

## Figures and Tables

**Table 1:** AABP results due to the mutation of the Omicron variant close to the cleavage site, six SD2-FP regions, and four HR1-CH regions. Red dash lines are locations of S1/S2 and S2′cleavages separating the 10 mutations. The first six mutations are labeled as group A, the second two mutations as group B, and the last four mutations as group C to facilitate the association of their relative positions with respect to the cleavage sites. AABP in units of electrons (e^−^). The light blue and tan color rows represent WT and OV respectively.

Models	Total AABP	NN AABP	NL-AABP	AABP (HB)	# NL AAs	Vol (Å^3^)	Surface (Å^2^)	PC*(e^−^)
WT D614	0.917	0.912	0.005	0.040	5	745.2	644.6	0.055
OV G614	0.908	0.907	0.002	0.042	3	640.3	564.5	0.961
WT H655	0.976	0.968	0.007	0.032	5	909.3	727.8	−0.827
OV Y655	0.971	0.965	0.006	0.032	5	944.5	745.3	−0.782
WT N679	1.022	0.956	0.066	0.081	3	624.6	533.9	0.833
OV K679	1.011	0.963	0.048	0.072	4	807.4	657.4	1.766
WT P681	1.117	1.064	0.054	0.064	5	872.2	757.9	2.862
OV H681	1.032	0.984	0.048	0.068	5	933.9	832.1	3.881
WT N764	1.130	1.008	0.121	0.137	6	959.5	775.1	1.043
OV K764	1.118	1.019	0.100	0.118	8	1211	986.4	1.181
WT D796	1.175	1.124	0.052	0.066	2	527.3	476.6	0.043
OV Y796	1.051	1.000	0.051	0.070	2	599.4	519.3	1.040
WT N856	0.935	0.894	0.041	0.069	3	609.9	554.0	0.783
OV K856	0.937	0.902	0.036	0.065	6	1081.0	822.5	1.801
WT Q954	1.148	1.008	0.140	0.152	7	1063.0	822.3	0.028
OV H954	1.146	1.008	0.139	0.155	7	1077.0	813.9	0.000
WT N969	0.938	0.907	0.031	0.052	5	774.1	624.5	0.188
OV K969	0.946	0.913	0.033	0.053	6	893.1	675.4	0.829
WT L981	0.898	0.893	0.005	0.036	5	960.1	754.9	0.090
OV F981	0.917	0.888	0.029	0.059	4	811.7	677.4	0.184

## Data Availability

The datasets used and/or analyzed during the current study available from the corresponding author on reasonable request.

## Supplementary Materials

The following supporting information can be downloaded at: https://www.mdpi.com/article/10.3390/microorganisms10101999/s1, Sections: S1. Electronic Structure, S2. Toward machine learning; Figure S1. Ball and stick figures of SD2-FP and HR1-CH showing the two cleavage sites marked in magenta, and Omicron variants mutations in the region of SD2-FP and HR1-CH are marked in green and orange, respectively. Red: O, blue: N, grey: C, white: H, yellow: S, Figure S2. Changes in intramolecular shape and size of specific mutations in the Omicron variant. Surface figures in different orientations for N856K in (a) and (a′), Q954H in (b) and (b′), N764K in (c) and (c′) and D796Y in (d) and (d′). The WT is on the left, while the OV is on the right. The surface of mutated sites is shown in magenta, the surfaces of NN and NL are shown in yellow and green, respectively. All NN and NL AAs are marked near to their surface in brown and black, respectively, Figure S3. Total density of states (TDOS) of the (a) SD2-FP model and (b) the HR1-CH model for both WT and OV, Figure S4. Partial density of states (PDOS) of the WT and OV amino acids for six sites of (a) SD2-FP model, and four sites of (b) HR1-CH model; Table S1. Data notation in machine-readable format for ML based on Table 1. The last digit is “0” for WT and “1” for the mutated type, Table S2. Machine-readable data notation in ML for the RBD-SD1 OV model. The last digit is “0” for WT and “1” for the mutated type.
