# Supplementary material for: Quantum Chemical Computation of Omicron Mutations Near Cleavage Sites of the Spike Protein
Source: Microorganisms. 2022 Oct 10;10(10):1999. doi: 10.3390/microorganisms10101999 (PMC9612061; doi:10.3390/microorganisms10101999)
Supplement: Supplementary file 1 [file microorganisms-10-01999-s001.zip › microorganisms-1948941-supplementary.pdf]

Content:

Sections

S1. Electronic structure

S2. Toward machine learning

Figures

Figure. S1. Ball and stick figures of SD2-FP and HR1-CH showing the two cleavage sites marked in magenta and Omicron variants mutations in the region of SD2-FP and HR1-CH are marked in green and orange respectively. Red: O, blue: N, grey: C, white: H, yellow: S.

Figure. S2. Changes in intramolecular shape and size of specific mutations in the Omicron variant. Surface figures in different orientation for N856K in (a) and (a'), Q954H in (b) and (b'), N764K in (c) and (c') and D796Y in (d) and (d'). The WT on the left while OV on the right. The surface of mutated sites is shown in magenta, surface of NN and NL are shown in yellow and green respectively. All NN and NL AAs are marked near to their surface in brown and black respectively.

Figure. S3. Total density of states (TDOS) of the (a) SD2-FP model, (b) HR1-CH model for both WT and OV.

Figure. S4. Partial density of states (PDOS) of the WT and OV amino acids for six sites of (a) SD2-FP model, and four sites of (b) HR1-CH model.

Tables

Table S1. Data notation in machine readable format for ML based on Table 1. The last digit is '0' for WT and '1' for the mutated type.

Table S2. Machine-readable data notation in ML for the RBD-SD1 OV model. The last digit is '0' for WT and '1' for the mutated type.

S1. Electronic structure

The simplest way to demonstrate the electronic structure is by presenting the total and partial density of states (TDOS and PDOS), a representation commonly adopted in condensed mater physics and/or materials science. In small molecules, they are usually presented in the form of energy levels close to the highest occupied molecular orbital (HOMO) and the lowest unoccupied molecular orbital (LUMO) separated by a small gap. This is obviously unpractical for large biomolecular systems with up to hundred thousand or more energy levels. In **Figure S3(a)** and **(b)**, we plot the TBOD of the two models SD2-FP (3654 atoms (WT) and 3681 atoms (OV)) and HR1-CH (3054 atoms (WT) and 3071 atoms (OV)) in each frame. The result is very interesting. For SD2-FP, the peaks in the TDOS are very close to each other with those OV at lower energy by about 0.15 eV and with a clear HOMO-LUMO gap of 1.60 eV. For SD2-FP, the structure of WT and OV are still very similar but those for OV are shifted to a lower energy with the same HOMO-LUMO gap of 1.60 eV. We set the energy scale of 0. eV or the HOMO level of WT for easy comparison. For HR1-CH, the general features are similar to SD2-FP except the peak separations are larger, of 0.71 eV. The HOMO-LUMO gap is 1.40 eV for WT and 1.70 eV for OV. What **Figure S3** shows is that the overall features of TDOS in WT and OV are quite similar. But

the TDOS in OV is lower in energy by about 1.5 eV from WT in HR1-CH compared to SD2-FP. Whether this feature is related to the relative positions of the cleavage sites S1/S2 and S2' is not clear at this time.

The TDOS in **Figure S3** is resolved into PDOS for each mutation in **Figure S4(a)** and **(b)**. While the peak positions in PDOS for WT and OV are still very similar, there are some interesting mutation-dependent features. For example, the area of PDOS below HOMO which accounts for the number of electrons is larger in OV than in WT, except for mutation D614G in SD2-FP model. This is consistent with the fact that these mutations increase the volume of AABPU with larger number of AAs, except in the case of mutation D614G. Although the mutation L981F in HR1-CH also decreases its volume by a small amount (see **Table 1**), so are the small difference in the area below HOMO in **Figure S4(b)**. Both these two mutations are farther away from the cleavage sites.

S2. Towards Machine Leaning

Another important direction that has not been addressed above is how to use the calculated data on AABPU in Machine Leaning (ML) applications in a creative way. ML depends on the existence of large amount of data points to facilitate the reliable prediction. Additionally, ML should be feed with high-accuracy input data representing all S-protein variants that have occurred or are expected to occur in different domains to obtain accurate prediction. The data should be calculate based on the realistic 3D structure of the proteins, rather than the traditional approach of using only the primary sequence. We anticipate using our high-accuracy data on AABPU as input to ML algorithm. In fact, the data that we have generated so far or that will be calculated is unique and cannot be found elsewhere. Because of this, we will be limited to using only our data to verify the accuracy of ML-predicted data. However, it should be mentioned that large data set are necessary to predict the potential new variants as well as the effect of the specific mutation and their consequences. Currently, we are at the stage of collecting accurate unique data that will be provide insight knowledge about the interatomic interactions in 3D including implicitly all AA-AA network in S-protein. We expect our project will provide at least 200 data points including the 20 data points in **Table S1** for 10 mutations based on the data from **Table 1.** Importantly, the key step is to provide specific data points in the form that can be read by machine as shown in the last column of **Table S1**. Furthermore, **Table S2** shows our preliminary results for the 16 mutations in Omicron in RBD-SD1 domain. The 56 data points from **Table S1** and **S2** enable us to start some preliminary tests such as using ¾ of the data to predict the other ¼. Our hopeful goal is using a smaller sample of highly accurate data based on our AABPU technique to predict the future mutations in S-protein. Of course, the success of this approach still needs to be tested and verified.

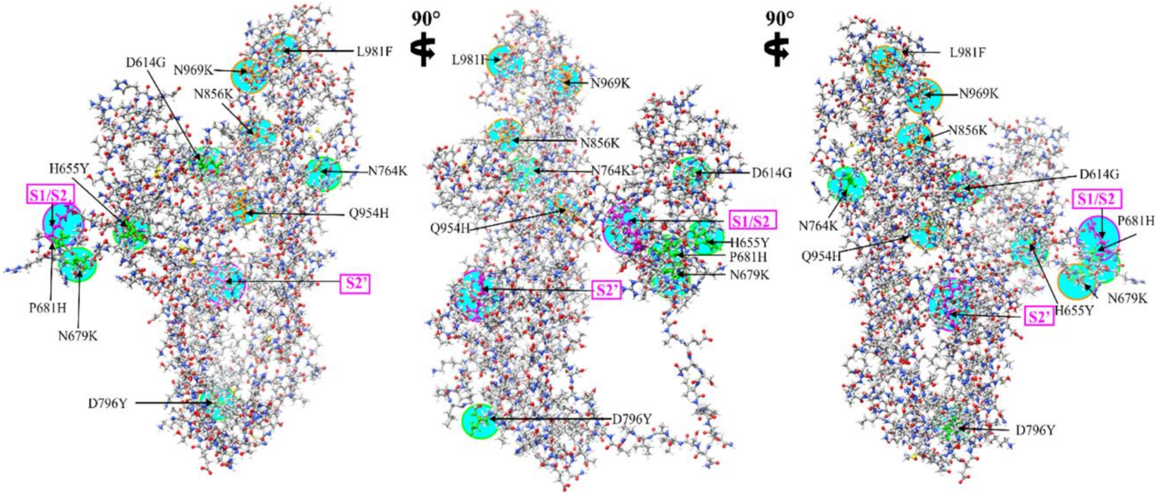

Figure. S1. Ball and stick figures of SD2-FP and HR1-CH showing the two cleavage sites marked in magenta and Omicron variants mutations in the region of SD2-FP and HR1-CH are marked in green and orange respectively. Red: O, blue: N, grey: C, white: H, yellow: S.

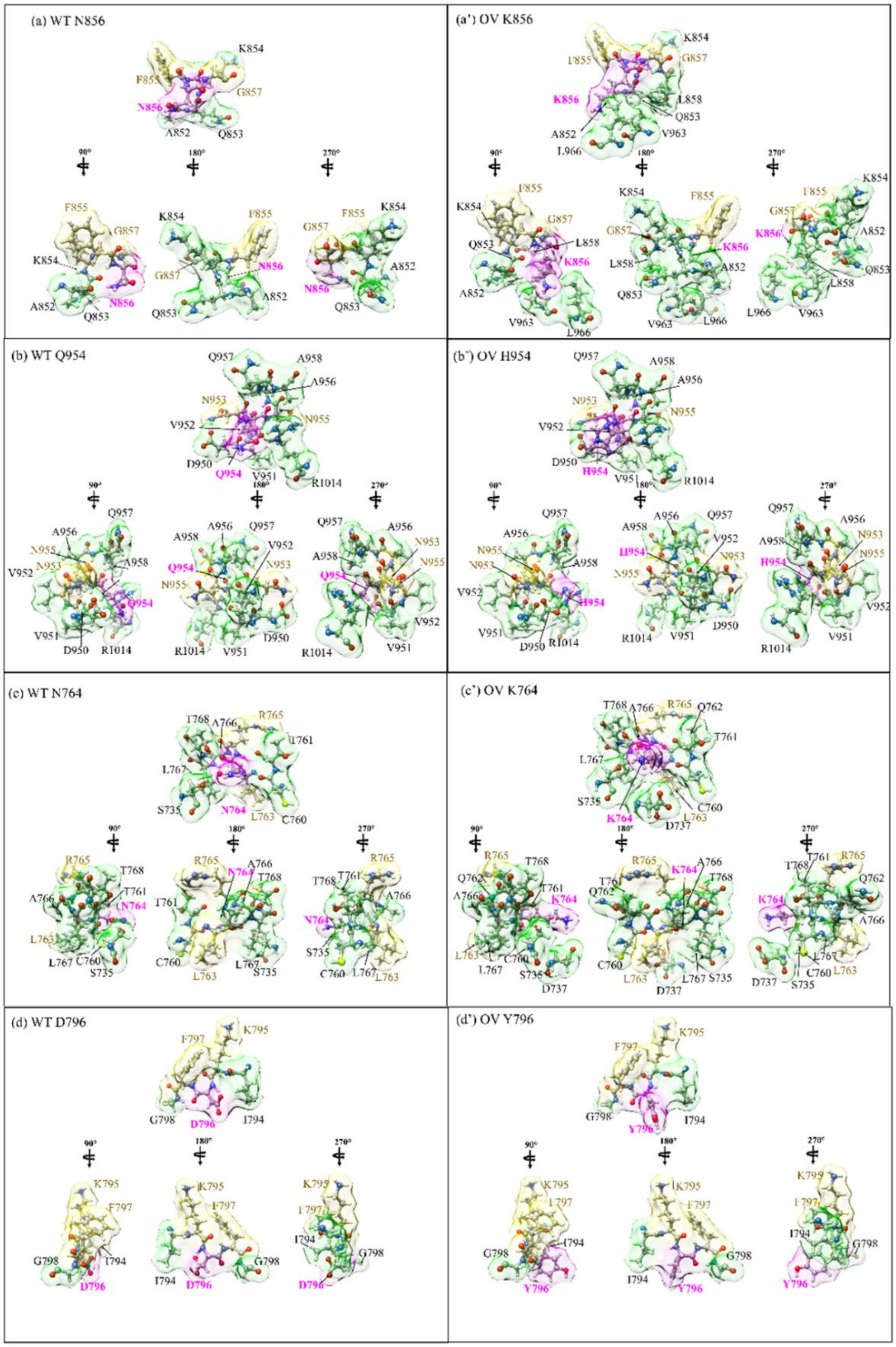

Figure. S2. Changes in intramolecular shape and size of specific mutations in the Omicron variant. Surface figures in different orientation for N856K in (a) and (a'), Q954H in (b) and (b'), N764K in (c) and (c') and D796Y in (d) and (d'). The WT on the left while OV on the right. The surface of mutated sites is shown in magenta, surface of NN and NL are shown in yellow and green respectively. All NN and NL AAs are marked near to their surface in brown and black respectively.

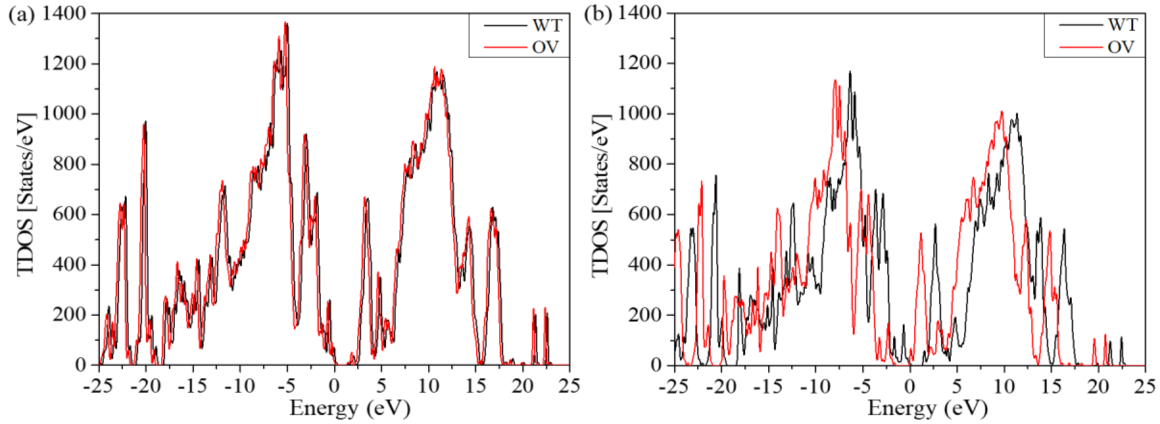

Figure. S3. Total density of states (TDOS) of the (a) SD2-FP model, (b) HR1-CH model for both WT and OV.

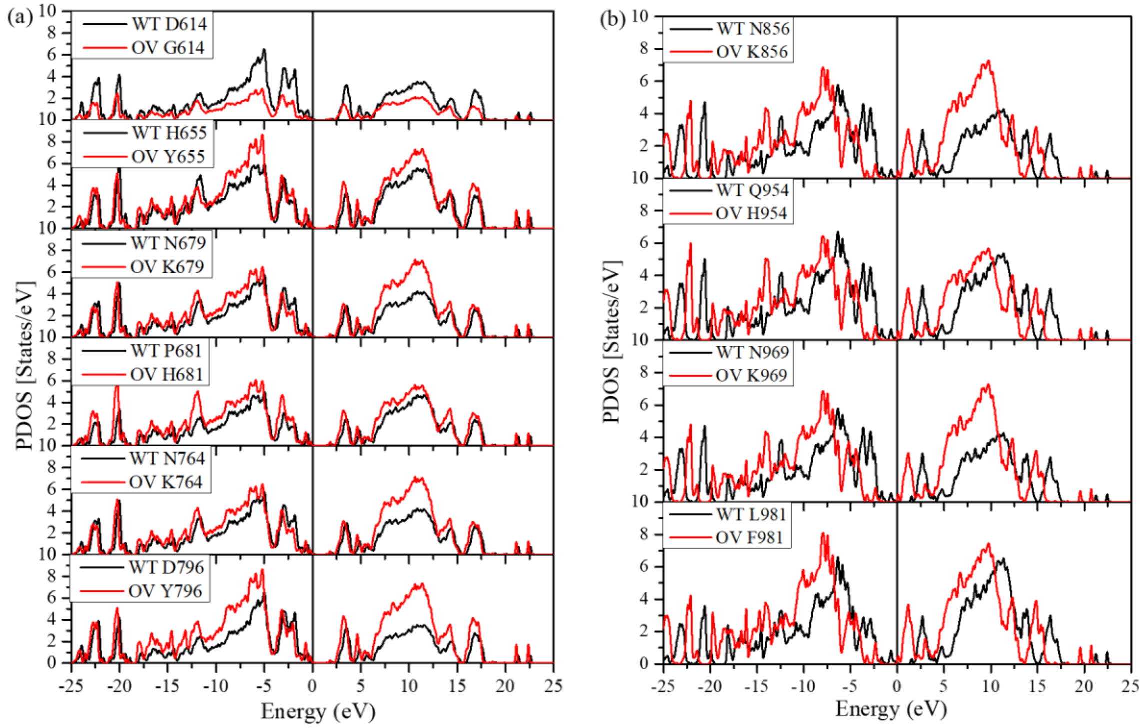

Figure. S4. Partial density of states (PDOS) of the WT and OV amino acids for six sites of (a) SD2-FP model, and four sites of (b) HR1-CH model.

Table S1. Data notation in machine readable format for ML based on Table 1. The last digit is '0' for WT and '1' for the mutated type.

Models	Total AABP	NN-AABP	NL-AABP	AABP (HB)	Data notation
WT D614	0.917	0.912	0.005	0.040	D614-0.917-0.912-0.005-0.040-0
OV G614	0.908	0.907	0.002	0.042	G614-0.908-0.907-0.002-0.042-1
WT H655	0.976	0.968	0.007	0.032	H655-0.976-0.968-0.007-0.032-0
OV Y655	0.971	0.965	0.006	0.032	Y655-0.971-0.965-0.006-0.032-1
WT N679	1.022	0.956	0.066	0.081	N679-1.022-0.956-0.066-0.081-0
OV K679	1.011	0.963	0.048	0.072	K679-1.011-0.963-0.048-0.072-1
WT P681	1.117	1.064	0.054	0.064	P681-1.117-1.064-0.054-0.064-0
OV H681	1.032	0.984	0.048	0.068	H681-1.032-0.984-0.048-0.068-1
WT N764	1.130	1.008	0.121	0.137	N764-1.130-1.008-0.121-0.137-0
OV K764	1.118	1.019	0.100	0.118	K764-1.118-1.019-0.100-0.137-1
WT D796	1.175	1.124	0.052	0.066	D796-1.175-1.124-0.052-0.066-0
OV Y796	1.051	1.000	0.051	0.070	Y796-1.051-1.000-0.051-0.070-1
WT N856	0.935	0.894	0.041	0.069	N856-0.935-0.894-0.041-0.069-0
OV K856	0.937	0.902	0.036	0.065	K856-0.937-0.902-0.036-0.065-1
WT Q954	1.148	1.008	0.140	0.152	Q954-1.148-1.008-0.140-0.152-0
OV H954	1.146	1.008	0.139	0.155	H954-1.146-1.008-0.139-0.155-1
WT N969	0.938	0.907	0.031	0.052	N969-0.938-0.907-0.031-0.052-0
OV K969	0.946	0.913	0.033	0.053	K969-0.946-0.913-0.033-0.053-1
WT L981	0.898	0.893	0.005	0.036	L981-0.898-0.893-0.005-0.036-0
OV F981	0.917	0.888	0.029	0.059	F981-0.917-0.888-0.029-0.059-1

Table S2. Machine-readable data notation in ML for the RBD-SD1 OV model (unpublished data). The last digit is '0' for WT and '1' for the mutated type.

**Models**	**Total AABP**	**NN-AABP**	**NL-AABP**	**AABP (HB)**	**Data notation**
WT G339	1.016	0.993	0.023	0.052	G339-1.016-0.993-0.023-0.052-0
OV D339	1.196	1.154	0.042	0.063	D339-1.196-1.154-0.042-0.063-1
WT S371	0.918	0.888	0.030	0.051	S371-0.918-0.888-0.030-0.051-0
OV L371	0.945	0.928	0.017	0.040	L371-0.945-0.928-0.017-0.040-1
WT S373	0.941	0.920	0.021	0.052	S373-0.941-0.920-0.021-0.052-0
OV P373	0.999	0.992	0.008	0.031	P373-0.999-0.992-0.008-0.031-1
WT S375	0.944	0.916	0.028	0.058	S375-0.944-0.916-0.028-0.058-0
OV F375	0.926	0.917	0.009	0.037	F375-0.926-0.917-0.009-0.037-1
WT K417	1.216	1.013	0.203	0.203	K417-1.216-1.013-0.203-0.203-0
OV N417	1.066	1.017	0.048	0.069	N417-1.066-1.017-0.048-0.069-1
WT N440	0.985	0.981	0.005	0.037	N440-1.066-0.981-0.005-0.037-0
OV K440	0.983	0.978	0.005	0.037	K440-0.983-0.978-0.005-0.037-1
WT G446	0.912	0.910	0.002	0.038	G446-0.912-0.910-0.002-0.038-0
OV S446	1.038	0.979	0.059	0.091	S446-1.038-0.979-0.059-0.091-1
WT S477	0.964	0.958	0.006	0.039	S477-0.964-0.958-0.006-0.039-0
OV N477	1.157	1.156	0.001	0.151	N477-1.157-1.156-0.001-0.151-1
WT T478	1.044	1.043	0.001	0.022	T478-1.044-1.043-0.001-0.022-0
OV K478	1.214	1.212	0.002	0.139	K478-1.214-1.212-0.002-0.139-1
WT E484	1.040	0.927	0.114	0.124	E484-1.040-0.927-0.114-0.124-0
OV A484	0.934	0.932	0.002	0.030	A484-0.934-0.932-0.002-0.030-1
WT Q493	1.060	0.973	0.087	0.106	Q493-1.060-0.973-0.087-0.106-0
OV R493	1.165	1.165	0.194	0.200	R493-1.165-1.165-0.194-0.200-1
WT G496	0.975	0.944	0.031	0.062	G496-0.975-0.944-0.031-0.062-0
OV S496	0.994	0.938	0.055	0.076	S496-0.994-0.938-0.055-0.076-1
WT Q498	1.120	1.073	0.047	0.054	Q498-1.120-1.073-0.047-0.054-0
OV R498	1.179	1.056	0.123	0.126	R498-1.179-1.056-0.123-0.126-1
WT N501	1.120	1.073	0.047	0.054	N501-1.120-1.073-0.047-0.054-0
OV Y501	1.034	0.942	0.092	0.104	Y501-1.034-0.942-0.092-0.104-1
WT Y505	1.058	0.974	0.084	0.104	Y505-1.058-0.974-0.084-0.104-0
OV H505	0.998	0.953	0.045	0.069	H505-0.998-0.953-0.045-0.069-1
WT T547	1.033	0.977	0.056	0.079	T547-1.033-0.977-0.056-0.079-0
OV K547	0.994	0.977	0.016	0.042	K547-0.994-0.977-0.016-0.042-1
